# The Child Oral Health Impact Profile—Short Form 19 Cross-Cultural Adaptation and Validity for the Portuguese Pediatric Population

**DOI:** 10.3390/jcm13164725

**Published:** 2024-08-12

**Authors:** Fanny Laborne, Vanessa Machado, João Botelho, Luísa Bandeira Lopes

**Affiliations:** Egas Moniz Center for Interdisciplinary Research (CiiEM), Egas Moniz School of Health and Science, 2829-511 Almada, Portugal; fannylaborne@gmail.com (F.L.); vmachado@egasmoniz.edu.pt (V.M.)

**Keywords:** oral health-related quality of life (OHRQoL), child oral health impact profile—short form 19 (COHIP-SF)

## Abstract

**Background/Objectives**: To better understand the impact of different oral conditions on children, several instruments are available to measure oral health-related quality of life (OHRQoL). To adapt and validate cross-culturally the Child Oral Health Impact Profile—Short Form 19 (COHIP-SF19) questionnaire to the Portuguese language. **Methods**: The COHIP SF-19 was translated and back-translated, and tested for its reliability and for psychometric properties in children who were aged between 8 and 17 years old. The COHIP-19-PT was tested for its internal consistency, construct validity, content validity, and test–retest reliability. **Results**: The COHIP-19-PT revealed good internal consistency (Cronbach’s alpha = 0.88) and test–retest reliability (interclass correlation = 0.78). The CFA analysis confirmed the structure of COHIP-19-PT. The first-order model showed an adequate fit: GFI = 0.878; CFI = 0.812; RMSEA = 0.083 (90% CI: 0.077–0.090). No invariance was found for the gender-based groups. The correlation between the sub-scales was also assessed, confirming significant correlations between all subdomains. **Conclusions:** The COHIP-19-PT is a valid and reliable scale for measuring children’s oral health-related quality of life.

## 1. Introduction

Oral health, being a crucial component of general well-being, significantly impacts an individual’s quality of life [1], and their chosen social roles [2]. The significance of personal perceptions of oral health becomes particularly crucial in assessing the social and psychological ramifications it engenders [3] and can play a decisive role in public oral health [4].

Oral health problems can negatively impact the quality of life of children and adolescents [5,6,7,8], especially since oral disease still has a high prevalence in children and dental caries remains a real public health problem worldwide [9]. Some oral conditions other than dental caries or dento-skeletal malocclusions can cause functional, aesthetic and psychological problems and affect the quality of life of children [10]. Thus, measuring children’s quality of life allows us to understand the impact of dental conditions on their health and wellness [11], and it allows us to facilitate the prioritization of individual health problems of these patients and the monitoring of treatment responses [5]. In the late 1980s and early 1990s, many measurement tools were developed and validated to assess the degree of impact of oral health on quality of life (QoL) in adults and the elderly. The most widely used are the Oral Health Impact Profile (OHIP), Oral Impact on Daily Performances (OIDP), Geriatric Oral Assessment Index (GOHAI), and Subjective Oral Health Status Indicators (SOHSI) [12]. However, the oral health quality of life assessment is age-specific. Compared to adults, children and adolescents have different perceptions of quality of life [13].

It was not until 2002 that pediatric instruments were developed to measure the oral health-related quality of life (OHRqOL) of children [11]. The three main questionnaires are the Child Perceptions Questionnaire (CPQ), the Child Oral Impacts on Daily Performances (C-OIDP), and the Child Oral Health Impact Profile (COHIP) [5,14].

The COHIP is a version adapted to children according to the criteria of the OHIP. This instrument allows one to measure the quality of life related to the oral health of children aged from 7 to 17 years. The original COHIP consists of 34 questions on five subscales: oral health, functional well-being, social and emotional well-being, school environment and self-image [15]. The short-form version includes 19 of the 34 items that are combined in three subscales: the oral health subscale, the functional well-being subscale and the social/emotional, school and self-image subscale [16,17,18].

The reduced version of the COHIP-SF was designed to receive a response from parents or children, and showed reliability and validity results comparable to the original version [19]. It should be noted that the COHIP-SF has been translated into Dutch, Korean, Persina, Chinese, Japanese and Indonesian, being important and useful for international comparison. Therefore, and since this instrument is not validated in Portugal, this study aims to adapt and cross-culturally validate the self-assessment questionnaire on the impact of oral health on the quality of life of children, demonstrating the reliability of the content, its internal coherence, and the validity of the construction. 

## 2. Materials and Methods

### 2.1. Translation and Pilot Test of Portuguese Version of COHIP-19 (COHIP-19 PT)

This study was carried out in accordance with the 2013 revision of the 1975 Declaration of Helsinki after receiving approval from the Institutional Review Board (Ethics Committee of Egas Moniz, ID: 190/2, Monte da Caparica, Portugal). The original English version of the COHIP—SF was translated and adapted in accordance with standard guidelines [20]. The original COHIP instrument was adapted and translated into Portuguese by three certified translators. The translated version was evaluated with double blindness, and a back-translation into English was carried out to confirm the existence of any discrepancies between the original and translated instrument. A panel of dental researchers evaluated the questionnaire and resolved any inconsistencies between the translations. The Portuguese version of the COHIP-19 was tested in a pilot phase on a random sample of 50 children from Campo de Flores school (Caparica, Portugal) (approximately 10% of the estimated sample). For reliability analysis, all 50 participants were asked to complete the questionnaire twice, two weeks apart. The pilot study demonstrated that the Portuguese version of COHIP had adequate semantic and conceptual equivalence. The children in the pilot study sample were not included for the validation analysis of the COHIP-19-PT questionnaire.

### 2.2. Cross-Cultural Adaptation of COHIP-SF Questionnaire

The original COHIP-SF tool was created as a psychosocial tool to assess how oral health affects children’s quality of life. This 19-item instrument is divided into 3 subscales: oral health (items 1 to 5), functional well-being (items 6 to 9), and social/emotional well-being, school environment and self-image (items 10 to 19). Each item is rated using a 5-point Likert scale, as follows: 0 = “never”, 1 = “almost never”; 2 = “sometimes”; 3 = “fairly often”; and 4 = “almost all of the time”. 

To study the psychometric properties of the COHIP-SF and to calculate the total score, the scores of negatively-terminated items were inverted (17 of 19 items), but not the scores of the two positively-terminated items, in order to guarantee consistency between them. The total scores ranged from 0 to 76 after recording, with higher scores indicating a better OHRQoL, and lower scores indicating a bigger impact of oral health on the children’s quality of life.

### 2.3. Sociodemographic Variables

The COHIP-19-PT assessment was complemented by a questionnaire on socio-demographic issues that included the child’s age and sex.

### 2.4. Sample Size Calculation 

Considering the requirements of using a Confirmatory Factor Analysis procedure and the characteristics of the parameters/dimensions to be assessed in COHIP-19, a minimum sample of 450 participants was estimated.

### 2.5. Design and Participants

All applied questionnaires were performed online and self-administered. The cross-sectional study was conducted in Almada (Portugal), with a target population of children between 7 and 17 years of age, who attended Egas Moniz Dental Clinic (EMDC), and in two schools (1 private, 1 public) selected at random from the region, which has 60 educational public establishments, and 32 private. Regarding inclusion/exclusion criteria, we included children aged between 7 and 17 years, children with Portuguese as their mother language, and the absence of cognitive impairment or other chronic illnesses. And as exclusion criteria, we used age above or below the specified range, the presence of cognitive impairments or other chronic illnesses and the presence of oral pain. Participation was voluntary and anonymous for subjects who met the inclusion criteria. Parents or other legal guardians had to approve and sign a written, free, and informed consent form before the children could participate. 

### 2.6. Reliability

The COHIP-19-EN reliability analysis was carried out using reliability tests and internal consistency analysis. Internal consistency was assessed using Cronbach’s alpha coefficient (α) in the R Studio program version 1.1-1 (R Studio Team 2018), in the “ltm” package. The α coefficient of 0.70 was considered acceptable for the COHIP-19-EN items [21]. The test–retest reliability assessed the two participants’ medication scores using the intraclass correlation coefficient (ICC) in the “irr” package of R version 0.84.1 (R Studio Team 2018). ICC values were interpreted as follows: excellent (above ≥ 0.90), acceptable (0.80–0.89), poor (0.60–0.79) and non-existent (below 0.60) [22].

### 2.7. Descriptive Analyses and Construct Validity 

Descriptive analyses of the basic characteristics of the target participants and of the COHIP-19-EN items and subscales were carried out and presented as numbers and percentages (%), mean and standard deviation (SD), median, and interquartile range (IQR) with minimum and maximum values. All the descriptive analyses were carried out using the statistical software R Studio version 1.0.8 (R Studio Team 18) in the “dplyr” package. A statistical significance level of 5% was adopted for all inferential analyses.

The Confirmatory Factor Analysis was carried out in the “lavaan” package of the statistical program R SWtudio version 0.6-10 (R Studio Team 2018) and was used to calculate the factor loadings and the adequacy of the model for each sub-construct. Maximum-likelihood estimation (MLE) was applied to estimate the model, and the differences between the models were assessed using the chi-squared test (χ^2^) and the likelihood ratio test.

## 3. Results

### 3.1. Reliability of COHIP-19-PT

After accounting for all the participants included for reliability testing, 27 (54%) were female and 23 (46%) were male, with similar age ranges (female, 7 to 17 years vs. male, 8 to 17 years, with an average of 12.22 ± 3 years). The mean total score on the COHIP-19-PT questionnaire was 63.44 (range 28–76).

The overall internal consistency measured by the Cronbach’s coefficient was 0.88 (95% CI: 0.71; 0.95), with all the subscales showing values above 0.70 and between 0.70 and 0.91. Nominally, the functional well-being subscale showed excellent reliability (Cronbach = 0.91 (0.82; 0.96)) (Table 1). The other scales showed acceptable reliability (Table 2). Furthermore, the ICC analysis revealed an overall result of 0.78 (95% CI: 0.71; 0.95) (*p* < 0.001), while each subscale showed acceptable coefficient values.

### 3.2. Participant’s Description

The test and retest groups were applied two weeks apart, and involved a total of 50 children (approximately 10%), who completed the COHIP-19-PT questionnaire, with an average age of 12.2 years (±3.0), ranging from 8 to 17 years (11.9 ± 2.8 and 12.5 ± 3.2 for males and females, respectively). The validation phase included 450 children with an average of 12.1 years old (±2.4), equally balanced for female–male ratio (214 girls and 236 boys, 12.1 ± 2.3 vs. 12.2 ± 2.5, respectively). 

The mean score of COHIP-SF was 59.4 (±10.6) with a median of 61.0 (IQR = 13.0), ranging between 18 and 76 (Table 3). The highest mean COHIP-19-PT scores were observed for items 6, 11 and 16, with 3.5 (±0.9), 3.6 (±0.7) and 3.7 (±0.7), respectively. Items 12 and 17 had the lowest scores at 2.1 (±1.5) and 2.0 (±1.4), respectively (Table 2).

### 3.3. Construct Validity

#### Factor Validity

The CFA analysis confirmed the structure of COHIP-19-PT (Table 3). The first-order model showed an adequate fit: GFI = 0.878; CFI = 0.812; RMSEA = 0.083 (90% CI: 0.077–0.090). The COHIP-19-PT showed adequate reliability with a Cronbach’s α coefficient of 0.88 (95% CI: 0.71–0.95), indicating adequate psychometric properties.

### 3.4. Gender Invariance Measurement

According to the results of the multi-group CFA (Table 3), no invariance was found for the gender groups based on comparisons of the CFIs, χ^2^ or degrees of freedom in the unrestricted and restricted models examined.

### 3.5. Relationships between COHIP-SF Components

The assessment of correlation between the sub-scales was also carried out, confirming significant correlations between all subdomains (Table 4).

## 4. Discussion

The study’s findings indicate that the COHIP-19-PT has been successfully translated, cross-culturally adapted, and validated, thereby providing a means of measuring the quality of life related to oral health in children with sound psychometric properties, this being important and useful for international comparison. Additionally, the results show that the COHIP-19-PT exhibited satisfactory reliability and internal consistency in both its overall score and its three subscales.

The confirmation of this instrument’s efficacy aligns with the majority of scientific findings from its validation in various other languages [17,18,23,24].

The COHIP is one of the most frequently used OHRQOL indexes in children, and the average final score of the COHIP-19-PT (59.5) is the average of countries where the COHIP-19 has already been validated, such as Germany (60.7), China (62.2), and Arab countries (61.13). In contrast, countries such as Indonesia have a lower average score (57.8). In fact, countries with dental surveillance in the national health system have better quality of life [17,18,23,24].

In the broader landscape of Portugal, the provision of oral healthcare is predominantly carried out by private practices, with dental services being largely unavailable within the National Health Service [25]. This lack of access to professional medical and dental services disproportionately affects socioeconomically disadvantaged individuals, further exacerbating existing health disparities.

In 2008, the “dentist voucher” system was implemented to promote free oral health care for the target population of this study. Although this system is a valuable support mechanism, the number of dentists integrated into the National Health Service is insufficient to meet the population’s needs. Consequently, curative treatments outnumber preventive treatments, and there are regions in the country where these services are unavailable. Moreover, some treatments are not covered by the system.

The need for dental treatment in pediatric patients has yet to be adequately assessed. The only study on the prevalence of dental caries in children was conducted in 2015 by the Directorate-General of Health of Portugal. The study assessed 1326, 1309, and 1075 children aged 6, 12, and 18 years, respectively. However, the distribution of subjects across specific age groups has limitations in terms of characterizing the prevalence and severity of dental caries in the pediatric population. Epidemiological studies are complex and require trained and calibrated dentists, specialized equipment, and various time-consuming implementation strategies. To overcome these methodological difficulties and promote oral health, self-report questionnaires like the COHIP can collect self-perceived information from individuals, including functional and psychosocial data about the disease [16].

The COHIP questionnaire has several advantages that significantly offset its limitations when used as a self-assessment instrument. This instrument is designed to gather information on the subjective perceptions and experiences of children regarding their oral health care. This information can be used to enhance the understanding of the needs and perspectives of pediatric patients [16]. Furthermore, the instrument includes sub-dimensions that allow for individual assessments of various aspects of the child’s oral health, functional well-being, socio-emotional well-being, school environment, and self-image. By using this instrument in conjunction with other indices and the child’s clinical characteristics, dentists can identify the child’s motivation for maintaining good oral health during consultations.

The limitations of the COHIP-19-PT require attention. The COHIP-19-PT relies on subjective, self-reported measures, which may be subject to bias due to variations in sociodemographic characteristics and oral hygiene habits. However, 17 of the 19 items received negative scores, while only two items (12 and 17) had positive scores, and these two items scored the lowest. This can be attributed to the questionnaire’s sequencing. The questions with negative scores were answered from items 1 to 11 and items 13 to 16, and when children answered items 12 and 17, they may have unconsciously responded similarly to how they did in the preceding questions. Research shows that reading a series of questions can influence an individual’s response, especially when the questions have simple choice answers [19,26,27].

Various criteria can also be considered when discussing the results of this study’s sample. The final COHIP-19-PT scores were similar for female and male children, as in the results for each of the three subscales. Cultural differences in the interpretation of quality of life may also explain some variations compared with international studies [17,18,23,24], and so one might argue that the regular harmonization and updating of the questionnaire might be useful to ensure coherence and reproducibility. Notably, most of the questionnaires were answered without supervision from the dentist or online. Therefore, it is not possible to confirm the involvement of parents or legal representatives in the child’s final response, and the literature shows that parents’ perceptions may be different from those of their own children [28]. In the future, to overcome this possible bias, the questionnaire should be applied and answered in a school context or in the dentist’s office, after the consent of parents or legal representatives has been collected.

In conclusion, the COHIP-19-PT questionnaire showed acceptable reliability, internal consistency and construct validity. This tool presents unique characteristics that can be used to measure OHRQoL in future public health programs and research in Portugal.

## Figures and Tables

**Table 1:** Test–retest reliability using Cronbach’s α and intraclass correlation coefficient for the COHIP-19-PT.

	Cronbach’s α (95% CI)	ICC (95% CI)
COHIP-SF Total Score	0.88 (0.71, 0.95)	0.78 (0.64; 0.87)
Subscales		
Oral-health	0.70 (0.44, 0.87)	0.51 (0.27; 0.69)
Functional well-being	0.91 (0.82, 0.96)	0.84 (0.73; 0.91)
Social/emotional well-being, school environment and self-image	0.89 (0.75, 0.95)	0.80 (0.68; 0.88)

CI—Confidence interval; ICC—intraclass correlation coefficient.

**Table 2:** Descriptive statistics of the COHIP-19-PT scores (mean, standard deviation (SD), median and interquartile range (IQR), minimum and maximum).

	Mean (SD)	Median (IRQ)	Min–Max
COHIP-SF Total Score	59.4 (10.6)	61.0 (13.0)	18–76
Item 1	3.1 (0.9)	3.0 (1.0)	0–4
Item 2	2.6 (1.4)	3.0 (2.0)	0–4
Item 3	3.3 (1.0)	4.0 (1.0)	0–4
Item 4	3.1 (0.9)	3.0 (1.0)	0–4
Item 5	2.8 (1.1)	3.0 (2.0)	0–4
Item 6	3.5 (0.9)	4.0 (1.0)	0–4
Item 7	3.3 (1.1)	4.0 (1.0)	0–4
Item 8	3.4 (0.9)	4.0 (1.0)	0–4
Item 9	3.1 (1.0)	3.0 (1.0)	0–4
Item 10	3.0 (1.3)	4.0 (2.0)	0–4
Item 11	3.6 (0.7)	4.0 (0.8)	0–4
Item 12	2.1 (1.5)	2.0 (3.0)	0–4
Item 13	3.1 (1.1)	4.0 (2.0)	0–4
Item 14	3.7 (0.8)	4.0 (0.0)	0–4
Item 15	3.3 (1.1)	4.0 (1.0)	0–4
Item 16	3.7 (0.7)	4.0 (0.0)	0–4
Item 17	2.0 (1.4)	2.0 (2.0)	0–4
Item 18	3.4 (1.0)	4.0 (1.0)	0–4
Item 19	3.1 (1.2)	4.0 (2.0)	0–4
COHIP-19-PT Subscale			
Oral-health	15.0 (3.1)	15.0 (4.0)	4–20
Functional well-being	13.3 (2.6)	14.0 (3.0)	2–16
Social/Emotional well-being, school environment and self-image	31.1 (6.9)	32.0 (8.0)	8–40

**Table 3:** Model fit indices in the unifactorial model and configuration invariance by sex.

Description	χ^2^	df	χ^2^/df	CFI	GFI	RMSEA (90% CI)	ΔCFI	Δχ^2^	Δdf
Unifactorial model	615.889	149	4.13	0.812	0.878	0.083 (0.077–0.090)	-	-	-
Measurement invariance across sex									
Unconstrained	797.61	298	2.68	0.804	0.979	0.086 (0.079–0.094)	-	-	-
Model 1	851.64	314	2.71	0.789	0.978	0.087 (0.080–0.094)	0.015	54.03	16
Model 2	911.02	330	2.76	0.772	0.977	0.088 (0.082–0.095)	0.027	59.38	16
Model 3	911.02	330	2.76	0.772	0.977	0.088 (0.082–0.095)	0.000	0.0	0

χ^2^—chi-squared, df—degrees of freedom, CFI—comparative fit index, GFI—goodness of fit index, RMSEA—root mean square error of approximation, Δ—delta.

**Table 4:** Correlation between COHIP-19-PT subscale scores.

COHIP-SF-PT	Oral Health	Functional Well-Being	Social/Emotional Well-Being, School Environment and Self-Image
Oral health	-	0.454 ***	0.437 ***
Functional well-being	-	-	0.486 ***
Social/emotional well-being, school environment and self-image	-	-	-

The values are Spearman’s correlation coefficient (rho), *** *p* < 0.001 means statistical significance.

## Data Availability

Data are freely available throughout the Supplementary Materials related to this work.

## Supplementary Materials

The following supporting information can be downloaded at: https://www.mdpi.com/article/10.3390/jcm13164725/s1, Table S1: Original version of the COHIP-SF 19 and translation into Portuguese.
