# Supplementary material for: The Child Oral Health Impact Profile—Short Form 19 Cross-Cultural Adaptation and Validity for the Portuguese Pediatric Population
Source: J Clin Med. 2024 Aug 12;13(16):4725. doi: 10.3390/jcm13164725 (PMC11355575; doi:10.3390/jcm13164725)
Supplement: Supplementary file 1 [file jcm-13-04725-s001.zip › jcm-3047554-SI.pdf]

Table S1. Original version of the COHIP-SF 19 and translation into Portuguese

COHIP-SF19		COHIP-19-PT
**In the past 3 months, how often have you?**		**Nos últimos 3 meses, com que frequência te deparaste com as situações abaixo?**
Item 1	Had pain in your teeth/toothache	Tiveste dor nos dentes/dor de dentes
Item 2	Had crooked teeth or spaces between your teeth	Tiveste dentes tortos ou espaçados
Item 3	Had discolored teeth or spots on your teeth	Tiveste dentes descolorados ou manchados
Item 4	Had bad breath	Tiveste mau hálito
Item 5	Had bleeding gums	Tiveste sangramento das gengivas
Item 6	Had difficulty eating foods you would like to eat	Tiveste dificuldade em comer alimentos que gostarias de comer
Item 7	Had trouble sleeping	Tiveste dificuldade em dormir
Item 8	Had difficultly saying certain words	Tiveste dificuldade em dizer certas palavras
Item 9	Had difficulty keeping your teeth clean	Tiveste dificuldade em manter os teus dentes limpos
Item 10	Been unhappy or sad because of your teeth, mouth, or face	Te sentiste infeliz ou triste por causa dos teus dentes, boca ou rosto
Item 11	Missed school for any reason because of your teeth, mouth, or face	Faltaste à escola por qualquer razão por causa dos teus dentes, boca ou rosto
Item 12	Been confident because of your teeth, mouth, or face	Te sentiste confiante por causa dos teus dentes, boca ou rosto
Item 13	Felt worried or anxious because of your teeth, mouth, or face	Ficaste preocupado/a ou ansioso/a por causa dos teus dentes, boca ou rosto
Item 14	Not wanted to speak/read out loud in class because of your teeth, mouth, or face	Não quiseste falar/ler em voz alta numa aula por causa dos teus dentes, boca ou rosto
Item 15	Avoided smiling or laughing with other children because of your teeth, mouth, or face	Evitaste sorrir ou rir com outras crianças por causa dos teus dentes, boca ou rosto
Item 16	Been teased, bullied, or called names by other children because of your teeth, mouth, or face	Foste provocado, importunado ou outras crianças chamaram-te nomes por causa dos teus dentes, boca ou rosto
Item 17	Felt that you were attractive (good looking) because of your teeth, mouth, or face	Te sentiste bonito/a por causa dos teus dentes, boca ou rosto
Item 18	Felt that you look different because of your teeth, mouth, or face	Te sentiste diferente por causa dos teus dentes, boca ou rosto
Item 19	Been worried about what other people think about your teeth, mouth, or face	Ficaste preocupado/a com o que as outras pessoas pensam dos teus dentes, boca ou rosto

Responses are recorded as “never” = 0, “almost never” = 1, "sometimes" = 2, “fairly often” = 3, and “almost all of the time” = 4. In portuguese: “nunca” = 0, “quase nunca”= 1, “por vezes”= 2, “com bastante frequência”= 3 and “quase sempre”= 4.
